# Deucravacitinib in concomitant psoriasis vulgaris and bullous pemphigoid: A case of sustained dual disease control

**DOI:** 10.1016/j.jdcr.2026.08.046

**Published:** 2026-09-04

**Authors:** Rachel Daher, Alessandro Magnatta, Walter Volpi, Luca Sanna, Virginia Corti, Simone Landini, Irene Bonanni, Marta Donati, Alice Verdelli, Marzia Caproni

**Affiliations:** a Department of Dermatology, Università degli Studi di Firenze, Piazza di San Marco, Florence, Italy; b Section of Dermatology, Department of Health Sciences, University of Florence, Florence, Italy; c Department of Dermatology, University of Modena and Reggio Emilia, Modena, and Azienda Unità Sanitaria Locale - IRCCS di Reggio Emilia, Skin Cancer Centre, Reggio Emilia, Italy; d Department of Dermatology, Rare Skin Diseases Unit, P.O. Piero Palagi, A USL Toscana Centro, ERN-SKIN Member, University of Florence, Florence, Italy

**Keywords:** autoimmune blistering diseases, bullous pemphigoid, deucravacitinib, psoriasis, tyrosine kinase 2

## Introduction

Psoriasis vulgaris is a chronic immune-mediated dermatosis driven predominantly by the interleukin (IL)-23/Th17 axis. Bullous pemphigoid (BP) is an autoimmune subepidermal blistering disorder characterized by autoantibodies against BP180 and BP230. An association between psoriasis and BP has been increasingly recognized, with epidemiologic studies demonstrating that each condition increases the risk of developing the other.^1^^,^^2^

Management of concomitant disease is challenging, as therapies may inadequately control or exacerbate one of the conditions.^1^^,^^3^ Deucravacitinib, an oral selective tyrosine kinase 2 (TYK2) inhibitor that modulates IL-23, IL-12, and type I interferon signaling, is effective in psoriasis, but its role in autoimmune blistering diseases remains unclear.^3^ Herein, we report a case of longstanding psoriasis complicated by BP in which treatment with deucravacitinib resulted in marked improvement of both conditions.

## Case report

A 75-year-old woman with a history of plaque psoriasis was referred to our dermatology unit for worsening disease activity. Her medical history included hypertension, hypercholesterolemia, and chronic hepatitis B virus (HBV) infection managed with lamivudine. At presentation, she exhibited pruritic erythematous–desquamative plaques involving the trunk, abdomen, and lower extremities. Previous histopathologic examination had confirmed psoriasis vulgaris. Initial management consisted of high-potency topical corticosteroids and calcipotriol/betamethasone foam.

In 2021, she developed worsening psoriatic activity accompanied by new tense bullae and erosions. Histopathology showed features consistent with an urticarial phase of BP. Direct immunofluorescence demonstrated perivascular C3 and C1q deposition in the superficial dermis, while indirect immunofluorescence showed linear IgG binding to the epidermal (roof) side of the dermo-epidermal junction. A commercial enzyme-linked immunosorbent assay (enzyme-linked immunosorbent assay; positivity threshold <9 U/mL) demonstrated anti-BP180 at 5.6 U/mL (below the threshold) and anti-BP230 at 10.6 U/mL (positive). The epidermal-side reactivity, together with anti-BP230 positivity and the clinicopathologic picture, supported BP and argued against dermal-binding subepidermal diseases such as epidermolysis bullosa acquisita and anti-p200 pemphigoid; immunoblotting and antigen mapping were not performed. These findings established a diagnosis of BP concomitant with psoriasis vulgaris.

Following multidisciplinary assessment, low-dose methotrexate (7.5 mg weekly) with folic acid supplementation was initiated. Despite systemic and topical therapy, both psoriasis and BP exhibited an intermittently relapsing course. Methotrexate was well tolerated, without hepatic or hematologic toxicity, but was ultimately transitioned for inadequate efficacy rather than intolerance. In 2024, examination revealed vesicular and crusted lesions on the lower extremities consistent with active BP, while psoriasis remained moderate in severity, with a Psoriasis Area and Severity Index (PASI) of 12.8. Given inadequate disease control, biologic therapy with an adalimumab biosimilar was initiated. Although well tolerated, psoriasis progressively worsened and BP reactivated despite dose adjustment, indicating secondary inefficacy and prompting a therapeutic switch.

In February 2025, clinical evaluation demonstrated extensive psoriatic involvement consistent with severe disease (PASI 28.8) (Fig 1), together with a single sacral bulla corresponding to a Bullous Pemphigoid Disease Area Index activity score of 1; serial serology showed that anti-BP180 and anti-BP230 titers had normalized (1.9 and 2.2 U/mL, respectively; January 2025). Deucravacitinib 6 mg daily was initiated in April 2025. At early follow-up, psoriasis was moderate (PASI 7.2) (Fig 2, *A*) with erosive BP lesions on the back (Fig 3, *A*), and improved progressively (PASI 4.8 by July 2025) (Fig 2, *B*) while BP fluctuated transiently (Fig 3, *B*); an annular lesion prompted mycologic testing, which was negative.

**Fig 1:**
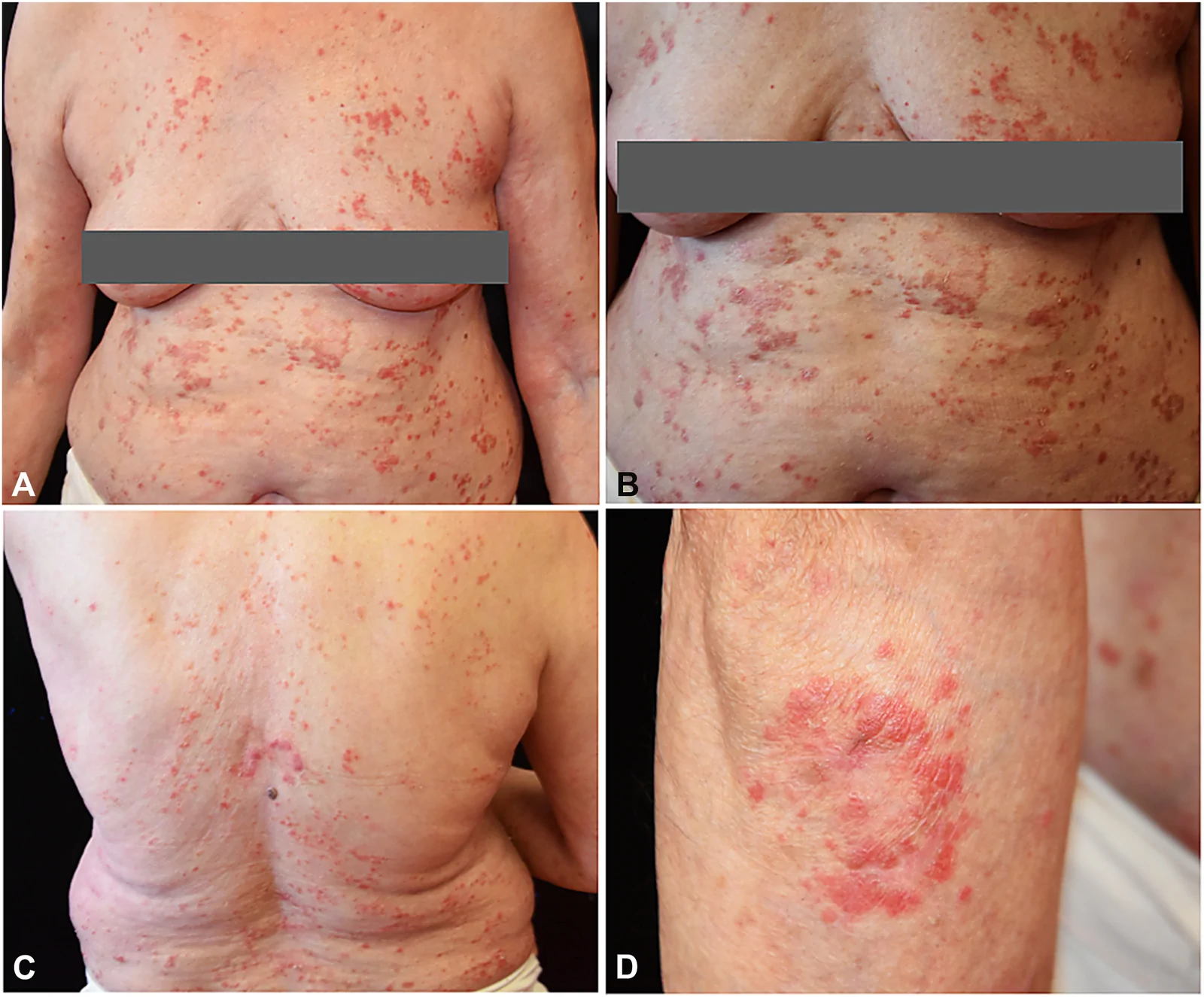
Psoriasis vulgaris prior to deucravacitinib initiation showing widespread erythematous papules and plaques involving the anterior trunk **(A)**, chest and abdomen **(B)**, posterior trunk **(C)**, and a representative plaque on the arm **(D)**. PASI was 28.8 (February 2025).

**Fig 2:**
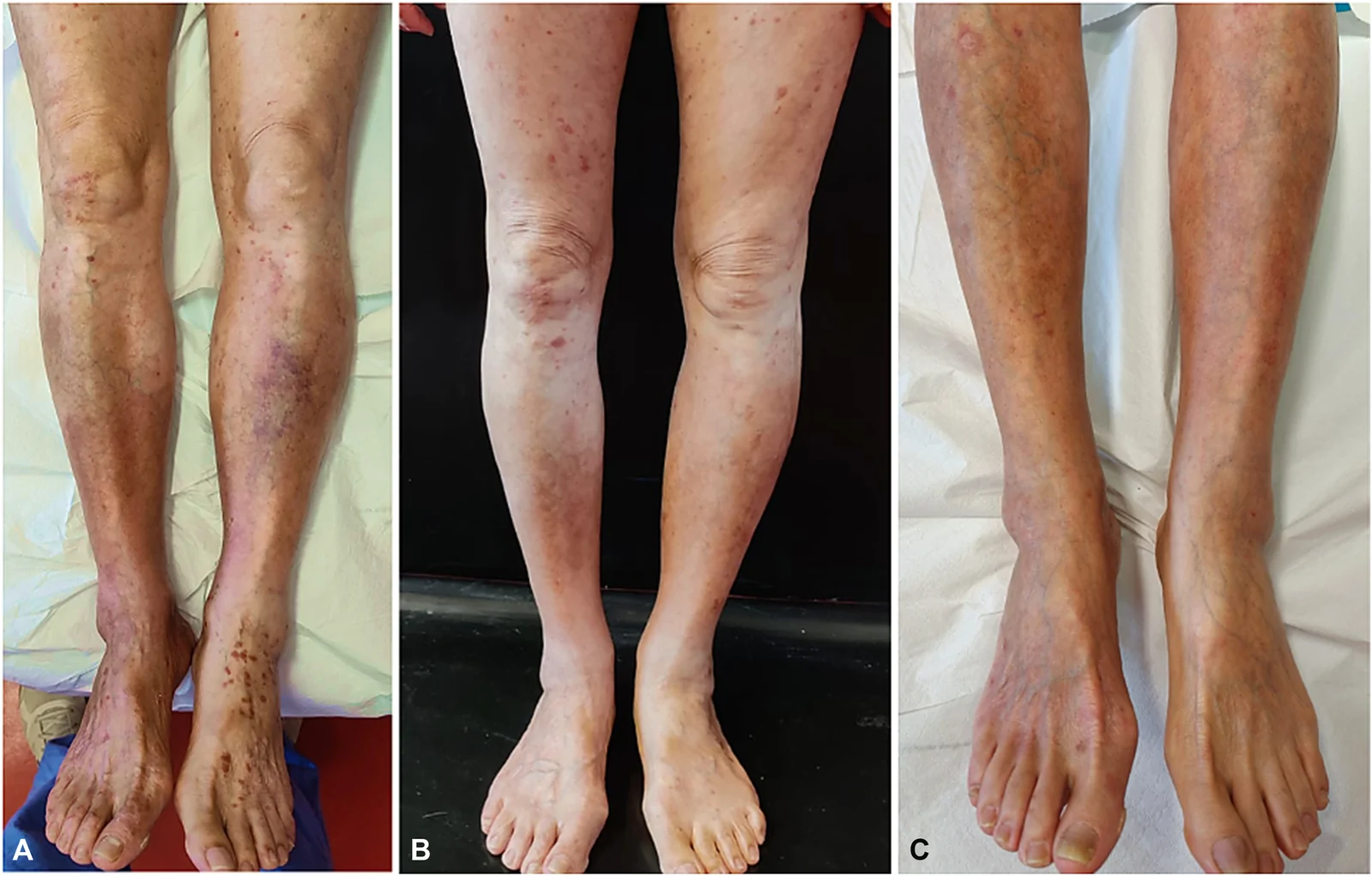
Clinical evolution of bullous pemphigoid lesions during deucravacitinib therapy. **A,** Erosive and crusted lesions on the upper back at early follow-up after initiation of deucravacitinib (June 2025). **B,** Transient inflammatory and erosive lesions on the trunk during follow-up (October 2025). **C,** Clinical resolution of lesions at follow-up during deucravacitinib therapy (January 2026).

**Fig 3:**
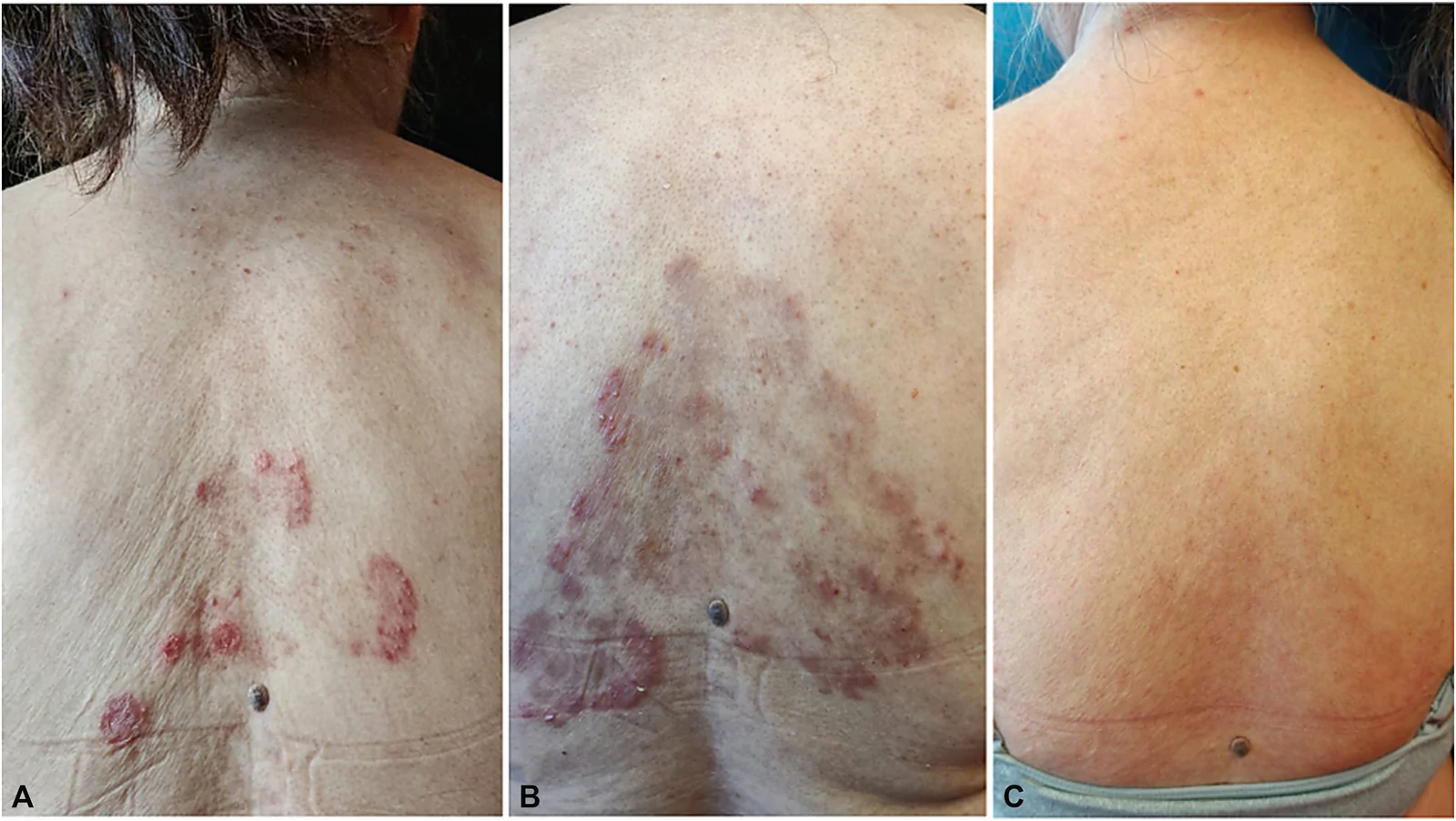
Clinical evolution of psoriasis during treatment with deucravacitinib. **A,** Psoriatic plaques on the lower extremities during early follow-up after initiation of deucravacitinib (June 2025; PASI 7.2). **B,** Partial clinical improvement with reduction in plaque burden during continued therapy (July 2025; PASI 4.8). **C,** Further reduction in lesion burden at the most recent follow-up (January 2026; PASI 2.4).

At the January 2026 evaluation, psoriasis was minimal (PASI 2.4) (Fig 2, *C*) and the Bullous Pemphigoid Disease Area Index activity score was 0 (Fig 3, *C*), consistent with clinical remission of both diseases according to current guidelines.^1^ Deucravacitinib was continued, with intermittent topical corticosteroids for occasional residual flares. At the most recent review, after approximately 12 months of therapy (April 2026), remission was maintained (PASI 2, Bullous Pemphigoid Disease Area Index 0). Throughout treatment, antiviral prophylaxis with lamivudine and periodic virologic and hepatic monitoring were continued without HBV reactivation. Peripheral eosinophil count, serum IgE, and on-treatment serology and immunofluorescence were not assessed.

## Discussion

BP is the autoimmune blistering disease most consistently reported in association with psoriasis.^1^^,^^2^ Epidemiologic studies support a bidirectional relationship, with psoriasis increasing the risk of subsequent BP and BP conferring an elevated risk of psoriasis; a systematic review and meta-analysis further confirmed an increased prevalence of psoriasis among patients with BP.^1^^,^^2^^,^^4^^,^^5^

The mechanisms underlying this association remain incompletely defined. Chronic psoriatic inflammation may promote structural and immunologic remodeling of the basement-membrane zone, facilitating antigen exposure and loss of tolerance through epitope spreading.^2^ Although BP has traditionally been considered a Th2-mediated disease, increasing evidence implicates Th17-related pathways, with elevated IL-17 in lesional skin and blister fluid and reduced experimental blistering upon IL-17A inhibition.^2^^,^^6^ Eosinophils are likewise central effectors, accumulating at the dermal–epidermal junction and releasing granule proteins, matrix metalloproteinase-9, and extracellular traps that promote dermal–epidermal separation under the influence of type 2 cytokines (IL-4, IL-5, IL-13).^7^ BP thus reflects a mixed type 2/type 17 cytokine milieu.

Given that TYK2 signaling lies upstream of IL-23-mediated Th17 activation, selective TYK2 inhibition may influence inflammatory pathways relevant to both diseases. Deucravacitinib selectively inhibits TYK2-dependent signaling downstream of IL-23, IL-12, and type I interferons, thereby targeting cytokine networks central to psoriasis pathogenesis 3; by attenuating IL-23–driven Th17 activity, it may also dampen the neutrophil and eosinophil recruitment and protease-mediated basement-membrane injury implicated in BP. Because deucravacitinib does not directly block IL-4, IL-5, or IL-13 signaling, any effect on the type 2/eosinophilic arm of BP would be indirect, mediated through these upstream pathways.

Management of concomitant psoriasis and BP remains challenging, as standard therapies for BP may exacerbate psoriasis and systemic immunosuppression may be limited by comorbidities.^2^ In this patient, deucravacitinib achieved a marked, objectively documented improvement in psoriasis (PASI 28.8 to 2) after failure of methotrexate and adalimumab, while BP—already of limited activity when treatment was switched—remained in sustained remission without relapse over 12 months. Although the fluctuating natural course of BP and the absence of on-treatment serology preclude a definitive attribution of the bullous outcome to deucravacitinib, the concurrent, durable control of both diseases in a treatment-refractory patient is clinically noteworthy.

Several features of this case merit emphasis. The patient's comorbid chronic HBV infection materially shaped therapeutic decision-making: conventional immunosuppressants and anti-tumor necrosis factor agents carry a recognized risk of HBV reactivation and required continuous antiviral prophylaxis with lamivudine and periodic monitoring of liver function and hepatitis B surface antigen. The comparatively selective, intracellular mechanism of TYK2 inhibition is attractive in this setting; although data on deucravacitinib in chronic HBV remain limited, prophylaxis and virologic surveillance were maintained throughout, without reactivation. Advanced age and cardiovascular comorbidities further favored an oral targeted agent over broader systemic immunosuppression.

Reports describing deucravacitinib in patients with concomitant psoriasis and BP remain scarce; however, inhibition of the broader Janus kinase/signal transducer and activator of transcription (Janus kinase/signal transducer and activator of transcription) pathway appears beneficial in BP, with successful use of JAK inhibitors such as upadacitinib and tofacitinib in patients with coexisting psoriasis.^8^^,^^9^ Conversely, deucravacitinib has also been implicated in triggering BP: new-onset BP was reported approximately 1 week after initiation in a psoriasis patient, hypothesized to reflect a TYK2-inhibition–driven shift of the Th1/Th17–Th2 balance toward relative Th2 predominance.^10^ That the same agent has been linked to both the onset and the control of BP underscores a context-dependent effect and supports monitoring psoriasis patients who begin TYK2 inhibitors for bullous eruptions.

In summary, epidemiologic data support a true association between psoriasis and BP, and mechanistic studies increasingly implicate Th17-related inflammation in its pathobiology.^2^^,^^4^^,^^5^ This case supports a potential role for selective TYK2 inhibition in patients with concomitant psoriasis and BP, particularly in treatment-refractory disease, while its single-patient design and incomplete on-treatment immunologic data warrant cautious interpretation. Further studies should define the role of TYK2 inhibition in autoimmune blistering disease and identify patients most likely to benefit.

### Declaration of generative AI and AI-assisted technologies in the writing process

None.

## Conflicts of interest

None disclosed.
